# Effect of Training on Visual Identification of High Frequency Oscillations—A Delphi-Style Intervention

**DOI:** 10.3389/fneur.2022.794668

**Published:** 2022-02-14

**Authors:** Aaron M. Spring, Daniel J. Pittman, Arsalan Rizwan, Yahya Aghakhani, Jeffrey Jirsch, Mary Connolly, Samuel Wiebe, Juan Pablo Appendino, Anita Datta, Trevor Steve, Neelan Pillay, Manouchehr Javidan, Morris Scantlebury, Chantelle Hrazdil, Colin Bruce Josephson, Cyrus Boelman, Donald Gross, Shaily Singh, Luis Bello-Espinosa, Linda Huh, Nathalie Jetté, Paolo Federico

**Affiliations:** ^1^Department of Clinical Neurosciences, University of Calgary, Calgary, AB, Canada; ^2^Hotchkiss Brain Institute, University of Calgary, Calgary, AB, Canada; ^3^Seaman Family MR Research Centre, Foothills Medical Centre, Calgary, AB, Canada; ^4^Department of Medicine, Queen's University, Kingston, ON, Canada; ^5^Department of Medicine, University of Alberta, Edmonton, AB, Canada; ^6^Department of Pediatrics, University of British Columbia, Vancouver, BC, Canada; ^7^Department of Pediatrics, University of Calgary, Calgary, AB, Canada; ^8^Department of Medicine, University of British Columbia, Vancouver, BC, Canada; ^9^Department of Neurology, Icahn School of Medicine at Mount Sinai, New York, NY, United States

**Keywords:** high frequency oscillations (HFO), generalizability theory, Delphi method, training, feedback, interrater reliability, intracranial electroencephalography (iEEG), epilepsy

## Abstract

**Objective:**

We examined the effect of a simple Delphi-method feedback on visual identification of high frequency oscillations (HFOs) in the ripple (80–250 Hz) band, and assessed the impact of this training intervention on the interrater reliability and generalizability of HFO evaluations.

**Methods:**

We employed a morphology detector to identify potential HFOs at two thresholds and presented them to visual reviewers to assess the probability of each epoch containing an HFO. We recruited 19 board-certified epileptologists with various levels of experience to complete a series of HFO evaluations during three sessions. A Delphi-style intervention was used to provide feedback on the performance of each reviewer relative to their peers. A delayed-intervention paradigm was used, in which reviewers received feedback either before or after the second session. ANOVAs were used to assess the effect of the intervention on the reviewers' evaluations. Generalizability theory was used to assess the interrater reliability before and after the intervention.

**Results:**

The intervention, regardless of when it occurred, resulted in a significant reduction in the variability between reviewers in both groups (*p*_*GroupDI*_ = 0.037, *p*_*GroupEI*_ = 0.003). Prior to the delayed-intervention, the group receiving the early intervention showed a significant reduction in variability (*p*_*GroupEI*_ = 0.041), but the delayed-intervention group did not (*p*_*GroupDI*_ = 0.414). Following the intervention, the projected number of reviewers required to achieve strong generalizability decreased from 35 to 16.

**Significance:**

This study shows a robust effect of a Delphi-style intervention on the interrater variability, reliability, and generalizability of HFO evaluations. The observed decreases in HFO marking discrepancies across 14 of the 15 reviewers are encouraging: they are necessarily associated with an increase in interrater reliability, and therefore with a corresponding decrease in the number of reviewers required to achieve strong generalizability. Indeed, the reliability of all reviewers following the intervention was similar to that of experienced reviewers prior to intervention. Therefore, a Delphi-style intervention could be implemented either to sufficiently train any reviewer, or to further refine the interrater reliability of experienced reviewers. In either case, a Delphi-style intervention would help facilitate the standardization of HFO evaluations and its implementation in clinical care.

## Introduction

High frequency oscillations (HFOs) have emerged as electroencephalographic (EEG) markers useful for localizing the source of seizure activity in patients with focal epilepsy (1–5).

However, findings amongst studies investigating HFOs have not been entirely congruent (1, 2, 6, 7), and its implementation in clinical practice, though increasing (7), has remained limited. Poor inter-rater reliability in HFO identification has been established (8) and recognized as a critical obstacle to the implementation of HFO analysis into routine clinical practice (9). The number of reviewers required to achieve highly reliable HFO evaluations was projected to be 17 using moderately trained reviewers (9), further limiting practical viability. At large epilepsy centers, producing highly reliable HFO evaluations would require extensive clinical hours; at smaller centers, there may not be sufficient clinicians available to complete this evaluation process. While establishing a baseline dataset using 17 similarly trained reviewers represents one possible method of overcoming poor inter-rater reliability, increasing the level of performance of a smaller pool of reviewers remains unexplored.

Paradoxically, expert opinion is used to identify a group truth, and ground truth is used to train experts. In the case of HFOs, the lack of a unifying definition of HFOs has been identified as one of the limiting factors in its implementation (10). The definitions established by experts vary, but are generally quite broad. A commonly cited definition describes HFOs as “spontaneous EEG patterns in the range of 80–500 Hz consisting of at least four oscillations that can be “clearly” distinguished from background […] characterized by a typical duration of 30–100 ms, an inter-event interval of at least 25 ms, and an amplitude of 10–100 μV” (11). Additionally, the short but complex nature of HFOs, and their low signal-to-noise ratio, make their clinical identification a unique challenge compared to seizures or spikes. In the case of seizures, the activity has a temporospatial progression, potentially has clinical correlation, and may also have some characteristic waveforms within it. Spikes alone are short, well-defined events that stand out from the baseline. HFOs, on the other hand, are more complex than spikes in that they constitute a larger number of cycles; are shorter and more localized than seizures; and are less distinguishable within the background. As such, it is clear that the identification of these HFOs, the establishment of a ground truth, and the ensuing training of experts is a more challenging task.

There is extensive research into training experts using a ground truth. Such training has been studied within the field of EEG analysis, and may take the form of intensive fellowships, streamlined online courses, or iterative feedback. Indeed, it has been shown that community neurologists perform significantly better on an EEG exam following a virtual EEG course (12). However, such methods inevitably rely upon a ground truth against which the performance of any user or candidate may be evaluated. In the case of HFO analysis, it has been established that no such ground truth yet exists (8), and conventional practice instead relies upon expert opinion.

Another approach to achieving consensus amongst a series of experts relies on the aggregation and feedback of their opinions or impressions. The Delphi method represents the original recognized method of achieving consensus from a panel of experts without any direct communication between the experts (13–15). The Delphi method consists of querying experts on a series of qualitative or quantitative items, collating the results, and providing feedback before querying them again. This process may be repeated until sufficient consensus has been achieved. It has been shown that after feedback has been provided, the variability between experts decreases, and strength of consensus increases (13, 14). This method has been adapted to other aspects of epilepsy research, such as for optimizing patient safety (16) or treatment initiation (17), but to our knowledge, it has never been used for the identification of HFOs or for evaluating electrographic signals in general. While it may enable a better agreement between experts on what constitutes an HFO, and would therefore improve interrater reliability, it would not replace the need for multiple experts to assess HFO data to establish a ground truth. Rather, it would reduce the number of experts required to review HFO data and achieve an opinion generalizable to the population of experts.

While obtaining a consensus on expert opinion can be easily evaluated with a Delphi method, it can be difficult to disentangle the effects of feedback from the effects of practice in a more task-based application. In the case of task-based training, the effects of feedback can be isolated by comparing an intervention group to a control group who does not receive an intervention. However, in the cases of smaller groups of subjects, delayed-intervention paradigms—in which one of the two groups merely receives the intervention at a later timepoint—have been used with success (18).

The overall objective of the present study is to evaluate the impact of reviewer experience and training on the inter-rater reliability of visually evaluated HFOs, capitalizing on both modified Delphi feedback and delayed-intervention control methodologies. The specific objectives of the first phase of the study are to establish a baseline inter-rater reliability for both experienced and inexperienced visual reviewers, and to project the number of reviewers that would be required to generate highly generalizable HFO evaluations. The second phase of the study aims to assess the role of consensus feedback in training visual reviewers, and its effects on inter-rater reliability and the number of reviewers required. Ultimately, the goal of this study is to establish a method of improving inter-rater reliability in the evaluation of HFOs, in order to facilitate the broader and more standardized implementation of HFOs in routine clinical care. Should the Delphi method prove effective in this application, it would reduce the number of epileptologists required to review a given EEG tracing and to identify HFOs in a generalizable manner, thereby reducing clinical hours and increasing the feasibility of clinical implementation at individual epilepsy centers.

## Methods

Nineteen board-certified epileptologists from three quaternary care epilepsy centers were recruited into the study as reviewers. Six had previously participated in an HFO study involving the evaluation protocol being implemented in the study (8, 9).

### Study Design

**Figure 1:**
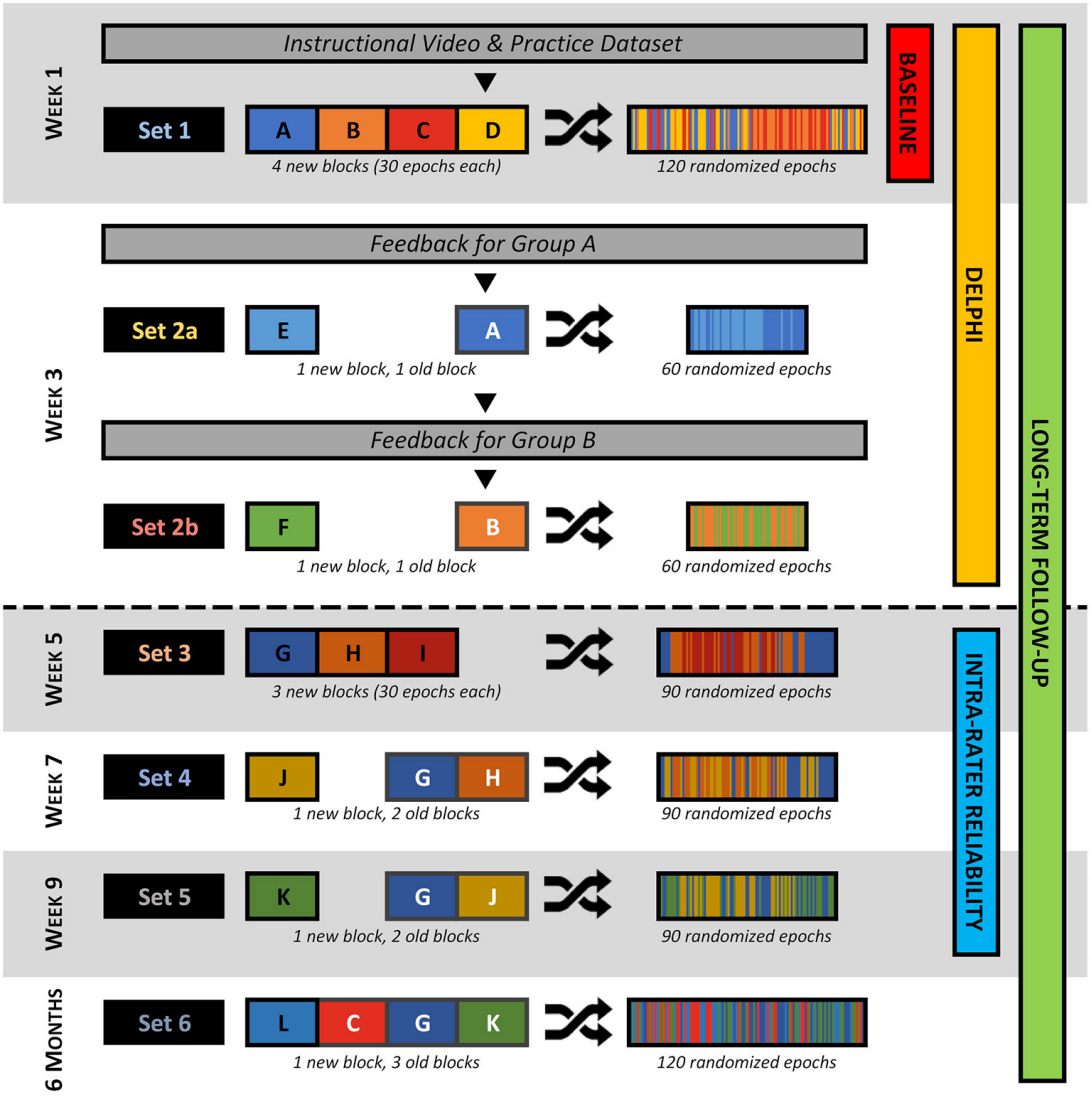
Diagram outlining the flow of the study. At the start, reviewers were given an instructional video and practice dataset. After this, each week, reviewers were presented with one or two datasets, each comprised of two to four “blocks” of data. These blocks may be new (black text and outline) or repeated from a previous dataset (white text and outline), but all consist of exactly 30 epochs (1 epoch per event type per patient) that were randomized. During Week 3, reviewers were presented with feedback before or after they complete Dataset 2a depending on which group they are randomized to. The corresponding phase(s) of the study are indicated on the right-hand side—those above the dashed line are included in the present work.

The study was designed to include four sequential phases, illustrated in Figure 1. In the first phase, reviewers were familiarized with the HFO evaluation program, and then completed a set of evaluations which was thereafter referred to as the baseline dataset. In the second phase, reviewers were provided with feedback of how their previous evaluations compared to those of their peers, before proceeding to complete a new series of evaluations; this enabled a *post-hoc* assessment of how their evaluations changed as a result of the feedback. In the third and final phases, reviewers evaluated data—with no intervention—at three sequential time points to assess intra-rater reliability, and again at a long-term follow-up to assess retention of the learning effect. The present paper describes the first two phases of the study.

#### First Phase—Baseline Dataset

All nineteen reviewers were recruited into the first phase of the study, to familiarize them with the evaluation program and to establish a baseline dataset. Reviewers were sent an instructional video and document to review at their convenience. An initial session was scheduled with each reviewer, wherein each reviewer was given an opportunity to complete evaluations on a practice dataset to further familiarize themselves with the evaluation program. The evaluations made on the practice dataset were not assessed, and no feedback on performance was given. Once the reviewer was comfortable with the evaluation process, they immediately began evaluation of the first study dataset (Dataset 1), which consisted of 120 epochs (described later).

#### Second Phase—Delphi Method Feedback

Fifteen of the 19 reviewers were recruited into the second phase of the study, designed to assess their responses to feedback on their performance, relative to that of their peers. One reviewer was excluded due to their involvement with the study design, and three reviewers were excluded due to inability to schedule the second phase. Each remaining reviewer was scheduled to participate in this phase of the study ~2–3 weeks following their first session, but at a time after all reviewers had completed their first session, as the feedback was generated from the performance of all participants.

These fifteen reviewers were randomized into two groups—one group (*n* = 8) received their feedback at the start of the second phase, while the other (*n* = 7) received their feedback in the middle of the second phase. In both cases, this feedback had been generated prior to the second phase, and therefore based only on performance in the first phase. Stratified randomization was used to control for experience and environment: reviewers were pairwise matched for these characteristics, and then randomized into opposite groups. Reviewers were blinded to the existence of multiple groups, and the team members constructing the feedback for the reviewers were blinded to the group assignments.

The dataset used in this phase of the study was divided into two equal halves, Datasets 2a and 2b, each consisting of 60 epochs. These each included 30 epochs that reviewers had seen previously during the first phase of the study, and 30 that were new and had never been seen previously by any of the reviewers. There were no overlapping epochs between Datasets 2a and 2b. The dataset was split to enable feedback to occur at different times, as described below, in order to disentangle the effect of learning from the effect of the feedback intervention itself. The combination of new and repeated epochs was to enable the effect of intra-rater reliability to be assessed more fully in future aspects of this study.

Reviewers in the early intervention group (Group EI) were presented with feedback at the start of their second evaluation session. The reviewers then proceeded to evaluate Dataset 2a and 2b consecutively, incorporating the feedback they had just received however they saw fit. Reviewers in the delayed intervention group (Group DI), however, evaluated Dataset 2a prior to receiving their feedback. Following the feedback, they were then able to evaluate Dataset 2b incorporating the feedback.

**Figure 2:**
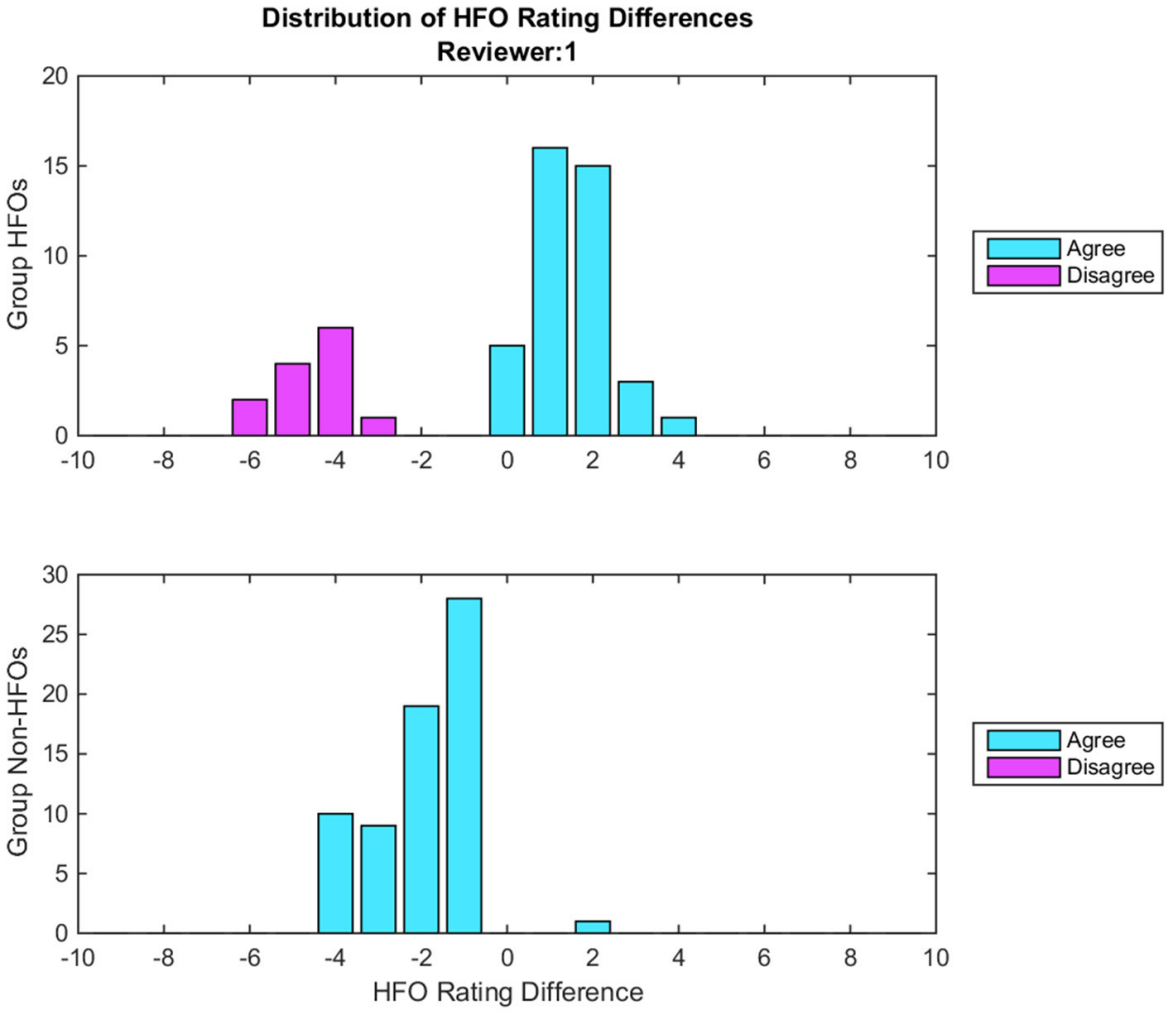
Example of histograms given during the feedback for one reviewer. Each histogram illustrates the distribution of the differences between the group consensus and the reviewer's HFO ratings. Cyan bars indicate epochs where there was agreement between the reviewer and the group consensus on whether an HFO is present, while magenta bars indicate disagreement. The zero bin indicates epochs where the reviewer's rating matches the group consensus. Positive bins indicate epochs that the reviewer had marked as more likely containing an HFO than the group consensus, where the magnitude equals the difference between the group consensus and the reviewer's rating. Negative bins indicate epochs that the reviewer had marked as less likely containing an HFO. The top histogram represents epochs marked by the Group DIs containing HFOs, while the bottom histogram reflects those marked by the Group DIs not containing HFOs. In this particular example, the top histogram illustrates that when the group marked an HFO, the reviewer agreed 75% of the time; this is indicated by 75% of the bars being cyan, and only 25% being magenta. In all cases of agreement, the reviewer was more certain than the consensus, typically by 1 or 2 confidence points. The bottom histogram here illustrates that when the group marked a non-HFO, the reviewer agreed 100% of the time, and was more confident than the group consensus in all but one instance, again typically by 1 or 2 confidence points. Overall, this reviewer rates both HFOs and non-HFOs confidently, but is generally less likely to identify an event as an HFO than the group on average.

The feedback reviewers received consisted of graphical, statistical, and descriptive information regarding their performance on the baseline dataset, relative to that of their peers. Two histograms illustrated how often they had agreed with their peers on HFOs and on non-HFOs, as well as how their confidence in their assessments differed from that of the group overall. They were also presented with an overall percentage of how often they agreed with the consensus HFOs, and how often they agreed with the consensus non-HFOs. This feedback was followed by 7 example epochs, differing according to the overall group assessment: almost certainly containing and not containing an HFO; likely containing and not containing an HFO; perhaps containing and not containing and HFO; and an effective toss-up. Finally, reviewers were shown 9 other epochs sequentially, each accompanied by details regarding the group's consensus, and their own rating of the epoch. An example of the feedback histograms is shown in Figure 2, while a complete example of the feedback is available in Appendix 1.

### Data Preprocessing

Eleven consecutive adult patients (mean age: 35.6 years) with drug-resistant focal epilepsy who were undergoing intracranial video-EEG monitoring for clinical purposes were recruited. Patient selection and data preprocessing were conducted per the methodology detailed in our previous study (8). All data were collected at a sampling rate of ≥1,000 Hz. Twenty minutes of iEEG data were selected, filtered (80-250 Hz), derived (bipolar or Laplacian), and normalized (sliding 1s root-mean-square). The filter was applied to isolate HFOs in the ripple band; references to HFOs made hereinafter refer specifically to those in the ripple band unless otherwise noted. Fast ripples (250–500 Hz) were not assessed in the present study. HFOs in the ripple band were identified without consideration for whether they occurred with or without overriding signals such as spikes or sharp waves.

Three types of events were algorithmically detected from the normalized data: candidate HFO events, low-threshold HFO events, and distractor events. Candidate HFO events contain an oscillation exceeding 3.1 standard deviations above the normalized signal amplitude; low-threshold HFO events contain an oscillation exceeding 2.3 standard deviations; and distractor events contain no such oscillations. For each event an epoch was constructed, consisting of both a 250 ms segment of filtered data (80–250 Hz), and a 3 s segment of unfiltered data. These epochs were ultimately what the visual reviewers evaluated for the presence or absence of HFOs.

In order to accommodate the study design, 11 blocks of 30 epochs were randomly generated from the collection of epochs assembled from the first 10 patients, for a total of 330 epochs. Each of the blocks contained exactly 1 epoch of each event type per patient (1 epoch per event type ×3 event types ×10 patients = 30 epochs). The blocks were then labeled and combined as illustrated in Figure 1 to generate six discrete datasets. The order of the epochs within each dataset was then randomized with restraints to prevent the development of context. The epochs were presented to reviewers during the evaluation sessions according to the study design, such that each reviewer evaluated the same epochs in the same order.

The practice dataset was assembled using 60 epochs randomly selected from the 11th patient (20 epochs per event type ×3 event types ×1 patient = 60 epochs). None of the epochs from the 11th patient's dataset were used for any analysis in this study.

### Evaluation Process

This study employed the same evaluation program and process as published in our previous work (8, 9).

#### Evaluation Program

**Figure 3:**
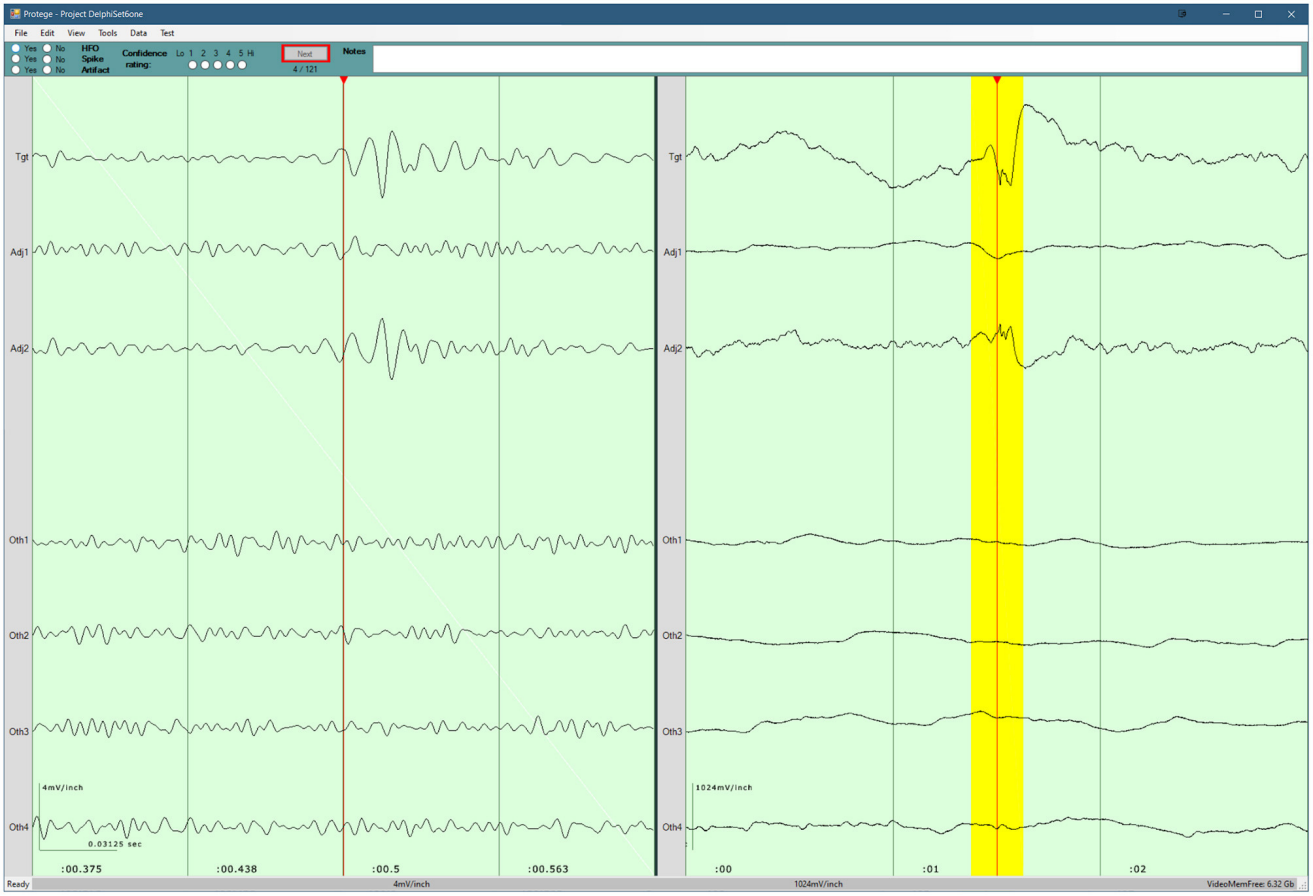
Screenshot of the program used for the visual review component of the study. Three seconds of raw data are shown in the right pane. 250 ms of filtered data are shown in the left pane, and the corresponding raw data are highlighted in yellow. The top pane contains the evaluation form for the current epoch, as well as the current progress. A detailed description of the evaluation program is available in our previous work (8).

For the evaluation of each dataset, the epochs were presented sequentially using the evaluation program developed in house, as illustrated in Figure 3. In particular, each epoch presentation included: 250 ms of filtered data (80–250 Hz) from a target channel containing one event of an undisclosed type (candidate HFO, low-threshold HFO, or distractor); 3 s of unfiltered data surrounding the epoch to provide temporal context. To provide spatial context, the display also presented the corresponding filtered and unfiltered data from the two nearest neighboring channels, and from four randomly selected other channels.

Each reviewer was instructed to identify HFOs that stood out from the surrounding baseline for at least 3 consecutive cycles, and to note the presence of any artifacts that they believed affected their determination of the presence of an HFO. Once an evaluation was complete, and the reviewer proceeded to the next epoch, the reviewer could not return to view and edit their evaluation for a previous epoch.

#### HFO Rating

Each reviewer registered an HFO rating for each epoch, on a scale from −5 to +5. The sign corresponded to the presence (+) or absence (–) of an HFO. The magnitude corresponded to the reviewer's confidence in the presence or absence of an HFO on a scale from 1 to 5, with 5 indicating complete certainty and 1 indicating complete uncertainty. As noted in our previous work, this rating scale affords the analysis of not only which epochs were marked as containing HFOs, but also the relative likelihood of each epoch containing an HFO, and the relative “stringencies” of the reviewers in identifying HFOs (9).

#### Deviation

For reviewers participating in the second phase of the study, it was also necessary to determine how much their ratings differed from those of the other reviewers in their group. For both the early and later intervention groups, a group consensus was calculated for each epoch as the mean rating given across the group. The raw difference between the group consensus and each reviewer's rating for each epoch yielded the raw deviation.

A root-mean-squared (RMS) deviation was also calculated for each reviewer: the raw deviations for the reviewer were squared, then averaged across a series of evaluations—namely, one or more evaluation sessions—and the square root was calculated. This yielded the RMS deviation for each reviewer at each time point (i.e., individual session or before/after intervention).

### Statistical Analyses

The first two phases of the study relied primarily on a combination of two different statistical models: analyses of variance (ANOVAs) and generalizability theory. Both approaches examine an output variable (in this case, the HFO evaluations) according to a number of other variables or effects. The latter approach has less widely been implemented, particularly in the HFO literature. In brief, generalizability theory consists of two distinct phases: a generalizability study (G-study) to compute variance components, as well as generalizability coefficients based on those variance components; and a decision study (D-study) to predict how changes in the sample sizes would affect the generalizability coefficients. In the present G-study, the HFO ratings made for each epoch by each reviewer were used as the G-study “measurement”. As such, the generalizability coefficients reflected how well the HFO ratings made by the group of reviewers would generalize to HFO ratings made by the universe of potential reviewers; in other words, it reflects inter-rater reliability for HFO ratings. In the present study, the 95% confidence intervals of the variance components were then used to approximate a liberal “confidence interval” (CI) for the generalizability coefficients and decision study projections. Generalizability theory is discussed further in our prior work (9), while both generalizability theory and the statistical analyses used in the present study are discussed further in Appendix 2.

#### First Phase—Baseline Dataset

Three generalizability and decision studies were undertaken in the first phase, all of which employed the same model as used in our previous study to estimate inter-rater reliability. The model accounts for the effects of the reviewer, epoch, and event type, as well as their interactions, in the determination of each HFO evaluation. The epoch—nested within the particular event type—was selected as the object of measurement because its corresponding generalizability coefficient represents inter-rater reliability (9). The model is expressed as:


$$\begin{array}{l} X_{rte}=\mu +\nu_{r}+\nu_{t}+\nu_{r\cdot t}+\nu_{e:\cdot t}+\nu_{r\cdot e:t} \end{array}$$

where *X*_*rdte*_ is the HFO rating given by Reviewer *r* to Epoch *e* of EventType *t;* μ is the grand mean HFO rating; and ν_α_ is the score effect for any arbitrary effect α.

The first generalizability study included data from all reviewers and was used to assess the inter-rater reliability of the reviewers. The corresponding decision study projected the number of similarly-experienced reviewers which would be required to achieve strong inter-rater reliability (>0.8). In order to compare experienced and inexperienced reviewers on inter-rater reliability and minimum number of reviewers required, these studies were repeated for two subsets of reviewers: those who had previously participated in an HFO study, and those who had not. The inter-rater reliability result for the experienced reviewers during this study was then compared to that obtained from them in the previous study.

#### Second Phase—Delphi Method Feedback

The effect of the intervention, a modified Delphi-style delayed-intervention paradigm, was also assessed. First, the effect was evaluated across all reviewers, regardless of when the intervention occurred. For Group DI (delayed intervention), the evaluations of Datasets 1 and 2a were compared with those of Dataset 2b. For Group EI (early intervention), the evaluations of Dataset 1 were compared to those of Datasets 2a and 2b.

An omnibus three-way ANOVA was performed using the squared deviation as the dependent variable, reviewer as the random effect, and group and intervention as the fixed effects. Subsequent two-way ANOVAs within each of the groups were performed using the squared deviation as the dependent variable, reviewer as the random effect, and intervention as the fixed effect.

The effects of intervention were visualized by plotting the RMS deviation of each reviewer, before and after the intervention. This facilitated the evaluation of the trends for each group and reviewer, and therefore the identification of potential outliers. Any outliers identified visually, and confirmed as being outliers by having *Z*-scores >3 or < −3, were removed, and both the omnibus and two-way ANOVAs were then repeated. One-way ANOVAs were also performed for each identified outlier, using the squared deviation as the dependent variable and intervention as the fixed effect.

Next, the effect of the intervention was disentangled from that of time, by repeating the analyses using session rather than intervention as the variable. This served to compare the evaluations of Dataset 1 with those of Dataset 2a for both Group DI—where no intervention had yet taken place—and Group EI—where the intervention had taken place between the evaluation sessions. The same three-way, two-way, and one-way ANOVAs were repeated, all substituting the intervention variable for a session variable. The effects of session were also visualized by plotting the RMS deviation of each reviewer for each session, and indicating the relative timing of the intervention.

Finally, the practical implications of the intervention were assessed using generalizability theory, employing the same model and object of measurement as used in the first phase of the study. The epoch generalizability (inter-rater reliability) using all 15 reviewers was calculated before and after the intervention. Decision studies were also performed, to project how the intervention affected the number of reviewers that would be required to achieve strong inter-rater reliability.

## Results

### Baseline Generalizability

**Figure 4:**
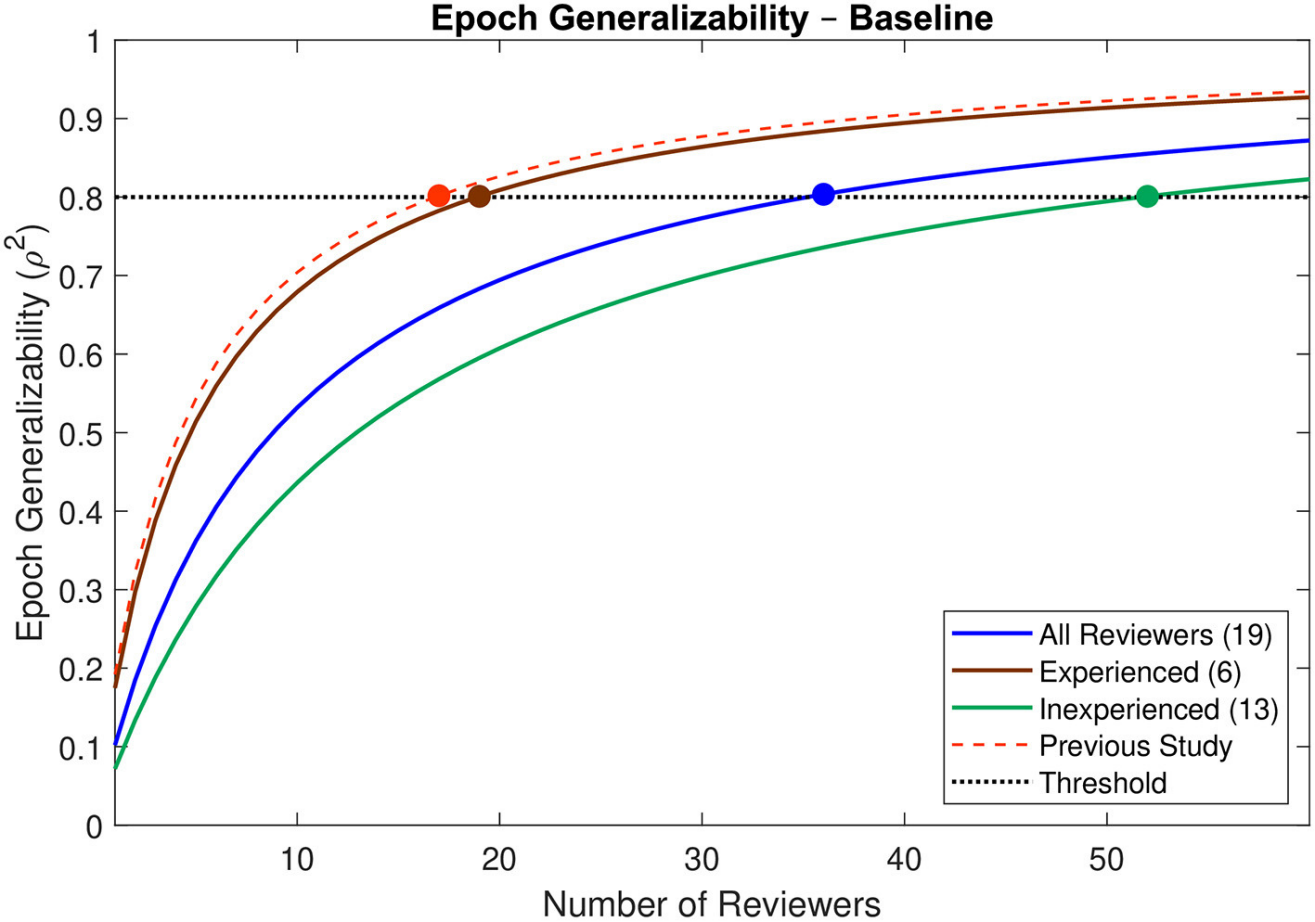
Decision study projections for epoch generalizability at baseline. Projections of the epoch generalizability based on the number of reviewers are presented for all 19 reviewers (blue line), for the six experienced reviewers (brown line), and for the 13 inexperienced reviewers (green line). The decision study projections from the previous study (9) are indicated by the dashed red line. The threshold of 0.8 is indicated by the dotted black line, and the number of reviewers projected to be required to achieve the threshold in each case is indicated by a colored marker.

The epoch generalizability coefficient for the baseline dataset (reviewed by all 19 reviewers) was 0.683 (CI: 0.559–0.759; Table 1a). Based upon the complete dataset, 36 reviewers (CI: 25–61) were estimated to be required to achieve strong generalizability (ρ^2^ > 0.8) (Figure 4). The dataset was then partitioned into two cohorts: one comprised of reviewers who had previously participated in an HFO study (*n* = 6), and the other of those who had not (*n* = 13). For the former, the epoch generalizability coefficient was 0.560 (CI: 0.376–0.676; Table 1b) and was projected to achieve the threshold of 0.8 with 19 (CI: 12–40) reviewers of similar characteristics. For the latter, the epoch generalizability coefficient was 0.501 (CI: 0.311–0.621; Table 1c), and was projected to exceed threshold with 52 (CI: 32–116) similarly inexperienced reviewers.

**Table 1:** Generalizability study details for each of the groups of reviewers.

**(a)**	**All 19 Reviewers**						
	**Facets**				**Object: Epoch**		
***α***	***σ*** **^2^(*α*)**	**CI *σ*^2^(*α*)**	***n*(*α*)**	***n*** **(*A*)**	***σ*** **^2^(*A*)**	**CI *σ*^2^(*A*)**	**Term**
*r*	0.425	0.000–1.039	19	19	0.0224	0.000–0.0547	Δ
*t*	n/a		3				
r*t	1.238	0.604–1.872		57	0.0217	0.0106–0.0329	Δ
***e:t***	**0.601**	**0.380–0.839**			**0.610**	**0.380–0.839**	**τ**
r*e:t	5.366	5.056–5.706		19	0.282	0.266–0.300	Δ,δ
**Generalizability coefficients**				**Term**	**Estimate**	**CI**	
Object variance component				σ^2^(τ)	0.610	0.380–0.839	
Relative residual variance				σ^2^(δ)	0.282	0.266–0.300	
Absolute residual variance				σ^2^(Δ)	0.327	0.277–0.388	
**Relative generalizability**				***ρ*** ^2^	**0.683**	**0.559–0.759**	
Absolute dependability				Φ^2^	0.651	0.495–0.752	
**(b)**	**Original 6 Reviewers**						
	**Facets**				**Object: Epoch**		
***α***	***σ*** **^2^(*α*)**	**CI *σ*^2^(*α*)**	***n*** **(*α*)**	***n*** **(*A*)**	***σ*** **^2^(*A*)**	**CI *σ*^2^(*A*)**	**Term**
*r*	0.572	0.000–1.848	6	6	0.0953	0.000–0.308	Δ
*t*	n/a		3				
r*t	1.094	0.00379–2.185		18	0.0608	0.00021–0.121	Δ
***e:t***	**1.268**	**0.676–1.859**			**1.268**	**0.676–1.859**	**τ**
r*e:t	5.987	5.356–6.737		6	0.998	0.893–1.123	Δ,δ
**Generalizability coefficients**				**Term**	**Estimate**	**CI**	
Object variance component				σ^2^(τ)	1.268	0.676–1.859	
Relative residual variance				σ^2^(δ)	0.998	0.893–1.123	
Absolute residual variance				σ^2^(Δ)	1.154	0.893–1.552	
**Relative generalizability**				**ρ** ^2^	**0.560**	**0.376–0.676**	
Absolute dependability				Φ^2^	0.523	0.303–0.676	
**(c)**	**Other 13 Reviewers**						
	**Facets**			**Object: Epoch**			
***α***	***σ*** **^2^(*α*)**	**CI *σ*^2^(*α*)**	***n*** **(*α*)**	***n*** **(*A*)**	***σ*** **^2^(*A*)**	**CI *σ*^2^(*A*)**	**Term**
*r*	0.496	0.000–1.202	13	13	0.0381	0.000–0.0925	Δ
*t*	n/a		3				
r*t	0.930	0.333–1.527		39	0.0238	0.00853–0.0392	Δ
***e:t***	**0.387**	**0.187–0.586**			**0.387**	**0.187–0.586**	**τ**
r*e:t	4.998	4.648–5.390		13	0.384	0.358–0.415	Δ,δ
**Generalizability coefficients**				**Term**	**Estimate**	**CI**	
Object variance component				σ^2^(τ)	0.387	0.187–0.586	
Relative residual variance				σ^2^(δ)	0.384	0.358–0.415	
Absolute residual variance				σ^2^(Δ)	0.446	0.366–0.546	
**Relative generalizability**				***ρ*** ^2^	**0.501**	**0.311–0.621**	
Absolute dependability				Φ^2^	0.464	0.255–0.616	

*In each case, the left pane provides an overview of variance components, and the right pane contains the calculated coefficients. The sets of columns in the left pane outline the variance components, (first column set) as well as the normalized variance components where the object of measurement is set to epoch (second column set). Confidence intervals are noted for variance components, normalized variance components, and generalizability coefficients. Generalizability studies are depicted for (a) all reviewers, (b) the six reviewers who have previously participated in similar studies, and (c) the other 13 reviewers. The object of measurement and the relative generalizability coefficient are highlighted in bold*.

### Delphi Effect on Ratings

#### Delphi—Intervention

The omnibus three-way ANOVA for intervention found a significant three-way interaction (*p* < 0.001) between the facets (group, intervention, reviewer), which precluded further interpretation of that statistical analysis (Table 2a). Subsequent two-way ANOVAs were performed within each of the two groups. Within Group DI, the main effects of intervention (*p* = 0.037) and reviewer (*p* = 0.006) were both significant, while the interaction between intervention and reviewer was not (*p* = 0.428) (Table 2b). Within Group EI, there was a significant two-way interaction (*p* < 0.001), precluding further interpretation (Table 2c).

**Table 2:** Summary of ANOVAs for intervention, reviewer, and group, including *F* statistics and *p*-values for each effect and their interactions.

**(a) Omnibus**	**All Reviewers**					
	***F***	***p***				
Intervention	10.74	0.006				
Group	2.17	0.165				
Reviewer (Group)	4.55	0.005				
Intervention*Group	0.69	0.419				
Intervention*Reviewer (Group)	**3.53**	**<0.001**				
**(b) Group DI**	**All Group DI**					
	***F***	***p***				
Intervention	**7.10**	**0.037**				
Reviewer	**10.22**	**0.006**				
Intervention*Reviewer	0.99	0.428				
**(c) Group EI**	**All Group EI**		**Excluding Outlier**		**Outlier (EI-1)**	
	***F***	***p***	***F***	***p***	***F***	***p***
Intervention	6.56	0.037	**22.95**	**0.003**	**83.15**	**<0.001**
Reviewer	3.52	0.060	**41.09**	**<0.001**		
Intervention*Reviewer	**6.67**	**<0.001**	0.64	0.697		

*Significant interactions precluding further interpretation are bolded and highlighted in orange. Significant effects are bolded highlighted in blue. (a) Three-way omnibus ANOVA including all reviewers. (b) two-way ANOVA for all reviewers within Group DI. (c) two-way ANOVA for all reviewers within Group EI, two-way ANOVA excluding Reviewer EI-1, and one-way ANOVA for Reviewer EI-1 only*.

**Figure 5:**
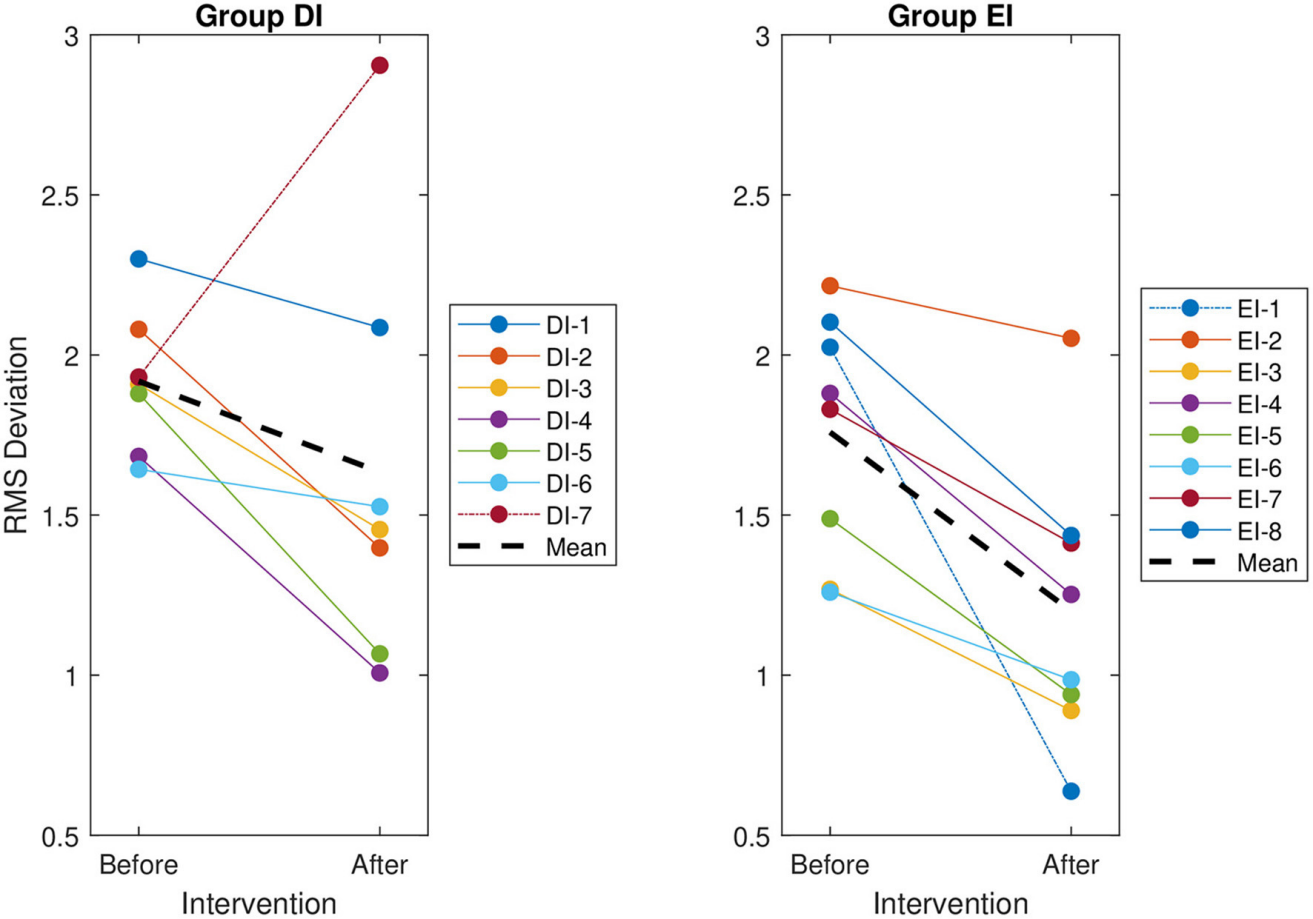
The root mean square (RMS) deviation of each reviewer's evaluations from the group mean, before and after the intervention. The RMS deviation for each reviewer is indicated by a solid line. The overall RMS deviation for each group is indicated by a thick dashed line. The identified special cases, indicated by a dot-dashed line, are DI-7 for Group DI—the only reviewer to deviate more from the group after the intervention—and EI-1 for Group EI—the only reviewer to improve visibly more than the other reviewers in the group.

Visual assessment of each reviewer's RMS deviation found that following the intervention, the deviations of Reviewer EI-1 improved substantially more than those of their peers (Figure 5). It was hypothesized that this single reviewer was driving into significance what would otherwise be an insignificant interaction, so the reviewer was classified as an outlier, removed from the group, and analyzed independently.

The ANOVA performed within Group EI, but excluding the outlier EI-1, found no significant interaction (*p* = 0.697). Both the main effects of intervention (*p* = 0.003) and reviewer (*p* < 0.001) were significant (Table 2c). The one-way ANOVA performed on the outlier EI-1 alone found a significant effect of intervention (*p* < 0.001).

It should also be noted that while 14 of the 15 reviewers improved following the intervention—that is, their RMS deviation from the consensus HFO ratings decreased—Reviewer DI-7 actually exhibited an increase in RMS deviation following the intervention (Figure 5). Notably, this reviewer was not treated as an outlier and was included in all statistical analyses.

#### Delphi—Session

Similarly, the omnibus three-way ANOVA for session also found a significant three-way interaction (*p* < 0.001) between the facets (group, session, reviewer), which precluded further interpretation of that statistical analysis (Table 3a). Subsequent two-way ANOVAs were again performed within each of the two groups. As in the intervention ANOVA within Group DI, the remaining two-way interaction between intervention and reviewer was not significant (*p* = 0.435), while the main effect of reviewer was significant (*p* = 0.008). However, the main effect of session was not significant (*p* = 4.14) within Group DI (Table 3b), where no intervention had taken place between the two sessions. Within Group EI, there was a significant two-way interaction (*p* < 0.001), precluding further interpretation (Table 3c).

**Table 3:** Summary of ANOVAs for session, reviewer, and group, including *F* statistics and *p*-values for each effect and their interactions.

**(a) Omnibus**	**All Reviewers**					
	***F***	***p***				
Session	6.38	0.025				
Group	4.24	0.060				
Reviewer (Group)	4.07	0.008				
Session*Group	2.93	0.111				
Session*Reviewer (Group)	**2.94**	**<0.001**				
**(b) Group DI**	**All Group DI**					
	***F***	***p***				
Session	0.77	0.414				
Reviewer	**9.39**	**0.008**				
Session*Reviewer	0.98	0.435				
**(c) Group EI**	**All Group EI**		**Excluding Outlier**		**Outlier (EI-1)**	
	***F***	***p***	***F***	***p***	***F***	***p***
Session	6.28	0.041	**11.94**	**0.014**	**41.97**	**<0.001**
Reviewer	2.90	0.091	**17.88**	**0.001**		
Session*Reviewer	**5.24**	**<0.001**	0.96	0.451		

*Significant interactions precluding further interpretation are bolded and highlighted in orange. Significant effects are bolded highlighted in blue. (a) Three-way omnibus ANOVA including all reviewers. (b) two-way ANOVA for all reviewers within Group DI. (c) two-way ANOVA for all reviewers within Group EI, two-way ANOVA excluding Reviewer EI-1, and one-way ANOVA for Reviewer EI-1 only*.

**Figure 6:**
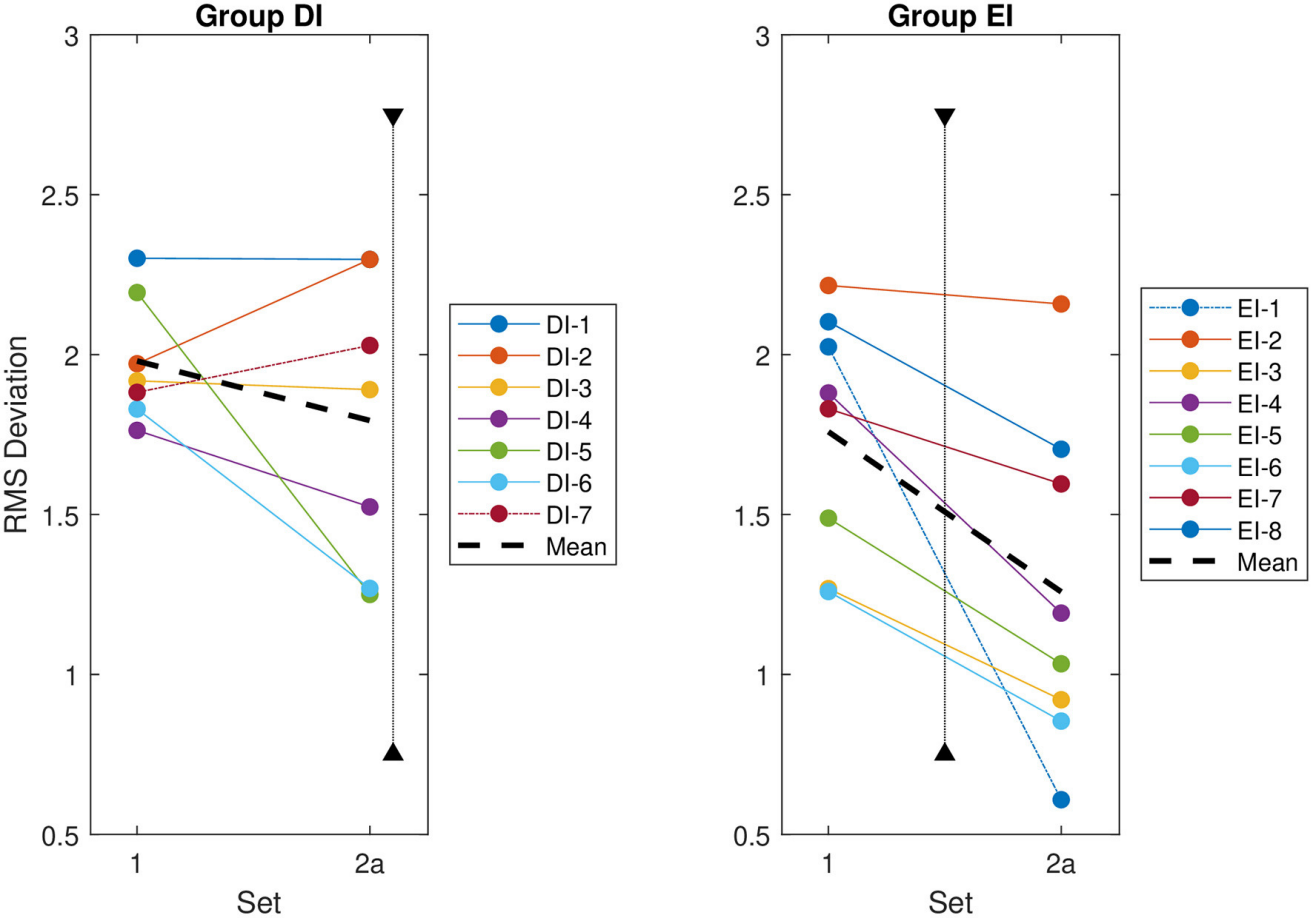
Root mean square (RMS) deviation of each reviewer's evaluations from the group mean for the first two datasets, regardless of whether an intervention has taken place. The RMS deviation for each reviewer is indicated by a solid line. The overall RMS deviation for each group is indicated by a thick dashed line. The special cases identified based on the intervention analysis—DI-7 for Group DI and EI-1 for Group EI—are indicated by dot-dashed lines. The timing of the intervention for each group is indicated by a vertical dotted line. This intervention occurs after Dataset 2a for Group DI, and between Datasets 1 and 2a for Group EI, as indicated on the figures.

The visual assessment was repeated and found again that the RMS deviation of Reviewer EI-1 improved more than that of their peers between the two sessions (Figure 6). As before, the analyses were then repeated independently for Reviewer EI-1 and the rest of Group EI.

The subsequent session two-way ANOVAs within Group EI paralleled the intervention ANOVAs. The ANOVA performed within Group EI, but excluding the outlier EI-1, found a non-significant interaction (*p* = 0.451). Both the main effects of session (*p* = 0.014) and reviewer (*p* = 0.001) were significant (Table 3c). The one-way ANOVA performed on the outlier EI-1 alone found a significant effect of session (*p* < 0.001).

### Delphi Effect on Generalizability

**Figure 7:**
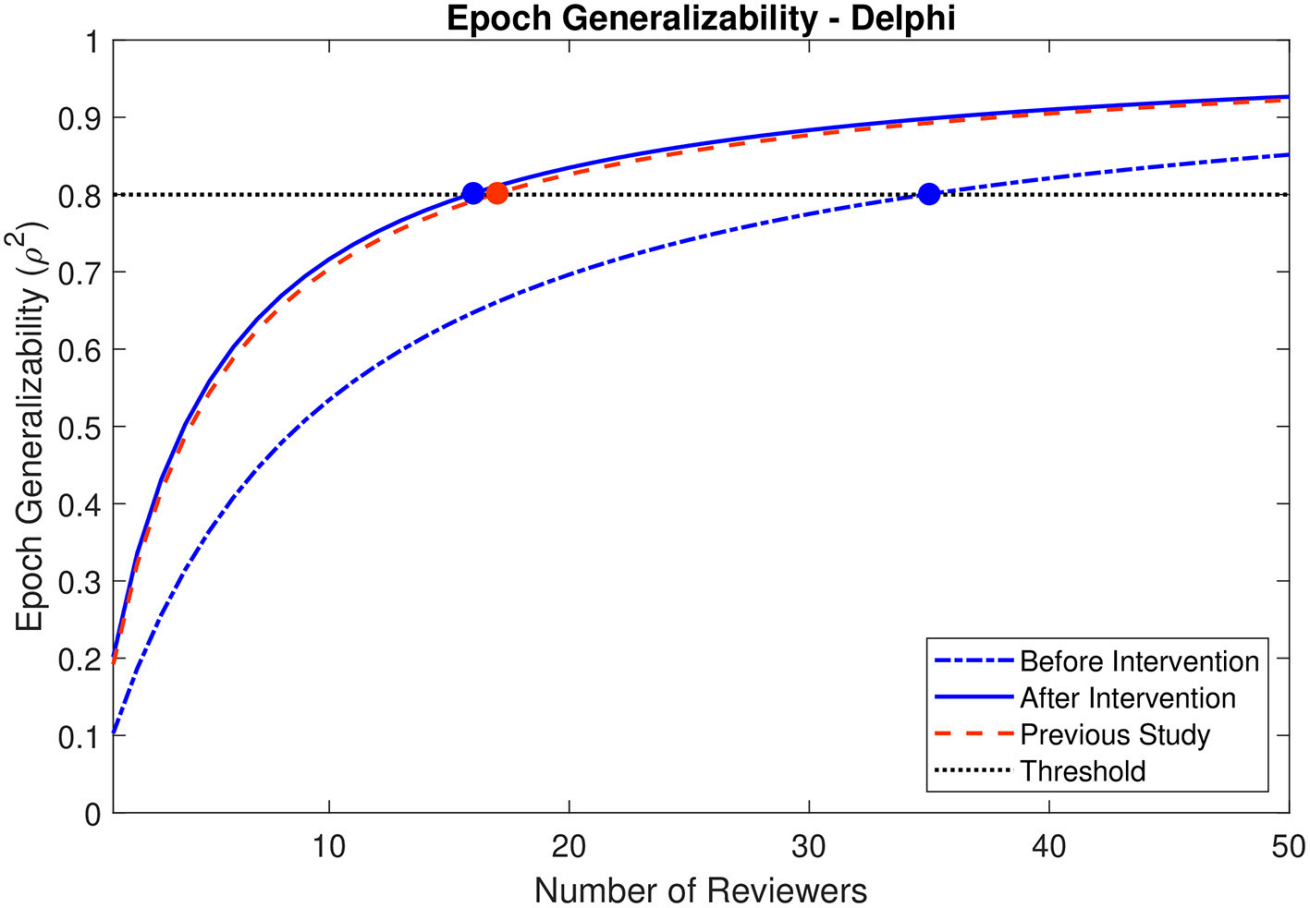
Decision study projections for epoch generalizability before and after the Delphi-style intervention. Projections of the epoch generalizability based on the number of reviewers are presented for all 15 reviewers before (dot-dashed blue line) and after (solid blue line) the intervention. The decision study projections from the previous study (9) are indicated by the dashed red line. The threshold of 0.8 is indicated by the dotted black line, and the number of reviewers projected to be required to achieve the threshold in each case is indicated by a colored marker.

For the epochs evaluated by the 15 reviewers participating in the second phase of the study, the epoch generalizability coefficients were calculated to be 0.633 (CI: 0.507–0.714) prior to the intervention, and 0.791 (CI: 0.694–0.847) following the intervention. The decision studies projected that 35 (CI: 25–59) reviewers would be required to achieve strong generalizability (ρ^2^ > 0.8) prior to intervention, whereas only 16 (CI: 11–27) reviewers would be required after the intervention (Figure 7).

Investigation of the four reviewers who stayed enrolled through to the second phase of the present study, who had also participated in a previous HFO study, revealed an epoch generalizability coefficient of 0.596 (CI: 0.435–0.702) prior to the intervention, and 0.709 (CI: 0.539–0.804) following the intervention. The corresponding decision studies projected that 11 (CI: 7–21) and 7 (CI: 4–14) reviewers would be required to achieve strong generalizability (ρ^2^ > 0.8) before and after the intervention, respectively.

For the 11 new reviewers who stayed enrolled through the second phase of the study, the epoch generalizability coefficient was 0.445 (CI: 0.254–0.570) prior to the intervention, and 0.672 (CI: 0.514–0.762) following the intervention. The corresponding decision studies projected that 55 (CI: 34–129) and 22 (CI: 14–42) reviewers would be required to achieve strong generalizability (ρ^2^ > 0.8) before and after the intervention, respectively.

## Discussion

The epoch generalizability was poor at baseline. It was projected that 36 reviewers, 19 experienced reviewers, or 52 inexperienced reviewers would be required to achieve strong epoch generalizability in the absence of any specific interventions. The Delphi intervention resulted in significantly more similar HFO ratings **in 14 of the 15 reviewers, necessarily increasing interrater reliability, and specifically improving epoch generalizability**. The corresponding projected number of reviewers required to achieve strong epoch generalizability improved to only 16 reviewers of any degree of prior experience—**even fewer than the 19 experienced reviewers required prior to intervention**, although with overlapping confidence intervals.

### Baseline Generalizability

The epoch generalizability of the baseline dataset was found to be poor (ρ^2^ = 0.683) using data from all 19 reviewers, despite a projection in our previous study that strong (ρ^2^ > 0.8) generalizability would be achieved with 17 reviewers (9). Indeed, the present study projected that 36 reviewers would be required—more than double the previous projections—and the liberal confidence interval (25–61) does not overlap with the previous point estimate. While this would initially appear to be incongruent, there are several key differences between the two studies. Most notably, the reviewers recruited into the previous study were generally more experienced in evaluating HFOs, while the majority of reviewers in the present study had no previous experience. Furthermore, the present study relied upon a much smaller dataset, comprised of data from several patients intermixed. This precluded reviewers from adapting their evaluations to the apparent frequency of HFOs in a particular patient, removing a substantial degree of context from their evaluations, thereby potentially increasing error and decreasing reliability. The previous study did not have such limitations, as the evaluations therein had been made sequentially for each patient, which allowed for some degree of context to the evaluations.

While it is difficult to quantify the effects of the lack of context or the small sample size on the generalizability, the inclusion of all participants from the previous study does allow for a more direct comparison between the two studies, and more importantly between experienced and inexperienced reviewers. In what is perhaps one of the more striking findings, the baseline epoch generalizability for the six experienced reviewers (ρ^2^ = 0.560) was similar to that for the 13 inexperienced reviewers (ρ^2^ = 0.501). Given that generalizability increases with sample size, this actually represents a substantial discrepancy, which is reflected in the decision study projections: 19 similarly experienced reviewers would be required to achieve strong generalizability, whereas 52 similarly inexperienced reviewers would be required. This not only highlights the marked discrepancy in interrater reliability between reviewers of different levels of experience, but also accounts for the majority of the discrepancy between the projections of the two studies. Indeed, the decision study projection in the previous study (17) is well within the liberal confidence interval in the present study using the same set of reviewers (12–40).

These findings further reinforce the need for unified training of reviewers, in order to overcome interrater variability, and produce highly generalizable HFO ratings. The second phase of the present study was undertaken to assess this factor through a Delphi-style feedback mechanism that could not only train the inexperienced reviewers, but could also refine the ratings of the experienced reviewers to become more concordant.

### Delphi-Style Intervention

The Delphi-style intervention was shown to significantly reduce the discrepancy between the HFO markings of most individual reviewers and the mean of all reviewers. This effect was robust and significant within both the early- and late-intervention groups.

This effect was also disentangled from the learning effect that may have been realized from repeating the same task over time. The early intervention group exhibited a significant improvement in HFO marking concordance at their second evaluation session, having received the intervention prior to the second session. The delayed intervention group, who had not yet received their intervention, did not show such improvement.

Two reviewers were handled as special cases. Reviewer EI-1 exhibited a greater improvement than their peers, causing an interaction between the intervention and reviewer effects, and was therefore excluded. After excluding EI-1, both the remainder of the group and the outlier alone were shown to have a significant decrease in RMS deviation following the intervention.

The second special case, Reviewer DI-7, was the only reviewer to show an increase in RMS deviation following the intervention. This reviewer was not excluded, and despite their inclusion the group analysis showed a significant decrease in RMS deviation across the group. This may have reflected a random variation; a change in behavior unrelated to the Delphi intervention; a shift in the mean score away from DI-7; or an over-correction in response to feedback. Upon closer inspection, it appears that the latter was the case. The feedback provided to DI-7 indicated that they were making HFOs more frequently than their peers, while their behavior after receiving the feedback was to mark HFOs much less frequently than their peers. This overshoot could be addressed by multiple iterations of Delphi-style feedback—making it clear to the reviewer than their previous adjustments were too large—or by revising the feedback format to optimize reviewer response. An iterative approach may also have the added benefit of further improving the performance of all reviewers relative to the group consensus, but would require interventions over a longer period of time.

### Delphi Effect on Generalizability

The observed decreases in HFO marking discrepancies across 14 of the 15 reviewers are encouraging: they are necessarily associated with an increase in interrater reliability, and therefore with a corresponding decrease in the number of reviewers required to achieve strong generalizability or interrater reliability. After the Delphi-style intervention, the projected number of reviewers required to achieve strong generalizability improved from 35 (CI: 25–59) to 16 (11–27). Notably, this projection—achieved from all reviewers, regardless of previous experience—is much more consistent with the projections achieved only using experienced reviewers prior to the intervention in this study (19 reviewers; CI: 12–40) or a previous study (17 reviewers) (9). Therefore, rather than limiting HFO identification to experienced reviewers, a Delphi-style intervention could be used to sufficiently train any reviewer.

The training effect was observed for the participants overall, as well as within the subsets of experienced and inexperienced reviewers. For the four experienced reviewers, the improvement in generalizability across the Delphi-style intervention corresponded with a decrease in the projected requirement of 11 reviewers to 7 reviewers. These findings alone have a number of further implications. It is clear that even experienced reviewers can benefit from a Delphi-style intervention, but that benefit may be affected by the confounding effects of the inclusion of inexperienced reviewers. The feedback presented in the study was an aggregate from all participants—both experienced and inexperienced—rather than from only the experienced reviewers, potentially increasing or decreasing the adaptations that the experienced reviewers had to make. A Delphi-style intervention performed exclusively with experienced reviewers may eliminate some of the additional noise from inexperienced reviewers and may further improve generalizability amongst more experienced reviewers. This may increase interrater reliability more rapidly amongst experienced reviewers. It is also likely that this study design marginally overestimates the projected number of reviewers due to a lack of context, or enforced similarity, compared to previous studies or real-world applications. In the present study, the epochs were randomized from multiple channels, patients, and time points, thereby stripping each epoch of any contextual similarity to the adjacent epochs. This results in a paradigm ideal for the assessment and training of reviewers but takes away key factors reviewers would use in real-world applications. While the exact effect on generalizability would be difficult to assess formally, it is illustrated by the discrepancy with the present projections and those of the previous study, which maintained more contextual similarity between epochs. This suggests it may be possible to achieve strong generalizability in a real-world application with even fewer than the projected 7 experienced reviewers following a Delphi-style intervention.

Additionally, repeated iterations of feedback may further reduce the interrater variability and decrease the projected reviewer burden. Ultimately, a set of highly generalizable HFO ratings could be reasonably generated in a large epilepsy center, either for clinical or research purposes, whether from 7 or even fewer experienced reviewers. In smaller epilepsy centers with fewer than the requisite 7 reviewers, this task would still not be feasible—implementation of HFOs in clinical practice in such centers would require either training an algorithm against these generalizable ratings, or further improvements in interrater reliability beyond the scope of the present study.

### Limitations and Future Direction

This study was limited to a single Delphi-style intervention, one of many Delphi interventions employed in previous applications within other fields. It is unknown whether iterative Delphi-style interventions would result in further improvement. Future studies incorporating multiple Delphi-style interventions would be required to evaluate the ongoing benefits of sequential interventions and could determine an optimal number of interventions for the improvement of interrater reliability. Such studies may also assess how frequently reviewers need feedback in order to retrain or recalibrate, to ensure their performance does not drift too far from consensus.

This type of Delphi-style intervention is not limited by location of reviewers. The feedback given is not geographically-specific and can be distributed anywhere with an internet connection. However, an intrinsic limitation of a Delphi-style intervention is that feedback to reviewers is dependent upon data collected from all participants. As such, there are temporal limitations with this type of set-up. All reviewers must complete any given stage of the Delphi study, and their results must be analyzed, prior to any reviewers embarking on the next stage of the study. This would complicate scheduling for large cohorts of reviewers, requiring either substantial administrative overhead or large latencies between study stages, the latter of which could limit the effectiveness of the intervention. Once a baseline set of HFO evaluations has been established by an adequate set of reviewers—projected to be 16 reviewers (CI: 11–27)—reviewers could then be trained against an established standard, rather than in a Delphi-style intervention. This training against an established standard is not subject to such temporal limitations, and could even occur in real-time, either in discrete training sessions or as an intensive intervention with online feedback.

As noted, generalizability theory does not intrinsically employ confidence intervals or hypothesis testing. This results in difficulty comparing results from different generalizability and decision studies, to assess whether observed changes are more likely meaningful or noise. Nonetheless, it is possible to calculate 95% confidence intervals of the variance components used in the generalizability theory calculations. Using variance components at the limits of the 95% confidence intervals, one can then generate an “upper limit” and “lower limit” for the generalizability coefficients, corresponding to a “lower limit” and “upper limit” decision study projection, respectively. These upper and lower limits do provide context to the point estimates, a liberal confidence interval of sorts, but they do not translate directly into a 95% confidence interval for the coefficients. As such, the “liberal confidence intervals” presented herein must be interpreted with caution.

The greatest limitation of the study is a result of the nature of HFOs themselves. This type of intervention trains reviewers based on the assessments of their peers, which is also subjective. It is unknown whether the criteria used by the reviewers—either before or after the intervention—reflect the features of HFOs most characteristic of the epileptogenic zone. Therefore, while this method may be used to generate a generalizable set of HFO markings that could then be evaluated as a marker of the epileptogenic zone, it could not yet be used to prospectively identify the epileptogenic zone.

Notably, the present study focuses on HFOs specifically in the ripple range (80–250 Hz). It does not assess fast ripples (250–500 Hz), distinguish between HFOs in different frequency ranges, or distinguish between ripples with or without spikes. Therefore, the results of this study apply only directly to HFOs in the ripple range, without consideration for the presence or absence of concurrent spikes. The choice of HFO type assessed in this study potentially affects the interrater reliability identified at the onset, and therefore on the necessity and clinical utility of its improvement. Nonetheless, the principle of improving interrater reliability through the Delphi method remains valid, and could be extrapolated to include other types of HFOs, other types of EEG waveforms (e.g., spikes or sharp waves), or even other electrographic signals. Further studies would be required to quantify the impact of a Delphi-style intervention on such signals.

## Conclusion

The interrater reliability of visually-identified HFOs is poor in experienced reviewers, and is even poorer in inexperienced reviewers. A Delphi-style intervention significantly decreases the discrepancy between HFO markings of individual reviewers and those of their peers, thus resulting in a corresponding increase in interrater reliability and decrease in the number of reviewers required to achieve strong generalizability.

The interrater reliability of all reviewers following a Delphi-style intervention, regardless of previous reviewer experience, is comparable to the interrater reliability of experienced reviewers without intervention. A Delphi-style intervention may be used to decrease the number of reviewers required to achieve strongly generalizable HFO markings, regardless of the level of experience of the particular reviewers. In the case of experienced reviewers, the Delphi-style intervention results in sufficiently high interrater reliability to produce a strongly generalizable set of HFO ratings with no more than 7 reviewers, enabling the practical implementation of generalizable evaluation of HFOs in large epilepsy centers for research purposes. Such an intervention provides a statistically reliable and practically meaningful tool for the improvement of interrater reliability, an essential step in the transfer of HFOs from research to routine clinical care.

## Data Availability Statement

The raw data supporting the conclusions of this article will be made available by the authors, without undue reservation.

## Ethics Statement

The studies involving human participants were reviewed and approved by University of Calgary, Conjoint Health Research Ethics Board. The patients/participants provided their written informed consent to participate in this study.

## Author Contributions

AS, DP, and PF contributed conception and design of the study. AS and PF performed the initial data collection. AS wrote the morphology detector and wrote the first draft of the manuscript. DP created the evaluation program. AS, DP, and AR performed the secondary data collection. YA, JJ, MC, SW, JA, AD, TS, NP, MJ, MS, CH, CJ, CB, DG, SS, LB-E, LH, NJ, and PF performed the primary data analysis. AS and AR performed the statistical analyses. AS, DP, AR, and PF interpreted the data. All authors contributed to manuscript review, and read and approved the submitted version.

## Funding

This work was supported by the Canadian Institutes of Health Research (MOP-230809). AS was supported by Alberta Innovates Health Solutions and the Canadian Institutes of Health Research.

## Conflict of Interest

The authors declare that the research was conducted in the absence of any commercial or financial relationships that could be construed as a potential conflict of interest.

## Publisher's Note

All claims expressed in this article are solely those of the authors and do not necessarily represent those of their affiliated organizations, or those of the publisher, the editors and the reviewers. Any product that may be evaluated in this article, or claim that may be made by its manufacturer, is not guaranteed or endorsed by the publisher.
