# Supplementary material for: Effect of Training on Visual Identification of High Frequency Oscillations—A Delphi-Style Intervention
Source: Front Neurol. 2022 Feb 14;13:794668. doi: 10.3389/fneur.2022.794668 (PMC8884138; doi:10.3389/fneur.2022.794668)
Supplement: Supplementary file 1 [file Data_Sheet_1.docx]

Supplementary Material

Effect of Training on Visual Identification of High Frequency Oscillations – a Delphi-Style Intervention

Aaron M Spring, Daniel J Pittman, Arsalan Rizwan, Yahya Aghakhani, Jeffrey Jirsch, Mary Connolly, Samuel Wiebe, Juan Pablo Appendino, Anita Datta, Trevor Steve, Neelan Pillay, Manouchehr Javidan, Morris Scantlebury, Chantelle Hrazdil, Colin Bruce Josephson, Cyprus Boelman, Donald Gross, Shaily Singh, Luis Bello-Espinosa, Linda Huh, Nathalie Jetté, Paolo Federico*

*** Correspondence:** Paolo Federico: pfederic@ucalgary.ca

# Appendix 1 – Feedback

The following is a feedback script, read out to an individual reviewer by a member of the research team. Each section was read out sequentially. Text in regular case was read to the reviewer, while text in italics describes performed actions. The reviewer was also provided with a textual version of this script.

## Introduction: Overall

You will now receive feedback from your previous HFO evaluation session. As there is no ground truth for HFO evaluations, apart from expert opinion, your responses are compared to the responses of the group overall. You may use this feedback to adapt your responses in future sessions, or not, as you see fit.

*[Visual HFO rating scale shown to reviewer]*

For every epoch, we used your HFO evaluation of present/absent and your confidence rating to generate an HFO rating. This rating is on a scale from 1 to 10, where 1 is confidently not an HFO (confidence of 5), 10 is confidently an HFO (confidence of 5), and 5 or 6 indicate a very low confidence rating (1) one way or the other.

For each epoch, we then calculated the average HFO rating across the 18 reviewers. As part of your feedback, we have prepared an overview of the difference between your rating and the group average for all of these epochs.

## Reviewer Performance: Overall Agreement

For epochs that the group average indicated an HFO was **present**, you agreed **75%** of the time.

For epochs that the group average indicated there was **no** HFO present, you agreed **100%** of the time.

## Introduction: Agreement by Rating

*[Histogram shown to reviewer (see Figure 1 for histogram shown to this reviewer)]*

This is pair of histograms illustrating the **difference** between your HFO rating and the group’s HFO rating for each epoch you evaluated. **Zero** indicates that you and the group evaluated the epoch with the **same** rating. Any **positive** number indicates that you marked the epoch as **more likely** an HFO than the group average, and any **negative** number indicates that you marked the epoch as **less likely** an HFO than the group average.

The top graph shows the epochs that were marked as being HFOs by the group overall. The cyan bars show the differences between you and the group for epochs where you agreed that an HFO was present. The magenta bars show the differences for epochs where you disagreed, and marked that no HFO was present.

Similarly, the bottom shows epochs that were marked by the group overall as not containing HFOs. The cyan bars show the differences between you and the group for epochs where you agreed that there was no HFO present. The magenta bars show the differences for epochs where you disagreed, and marked than an HFO was present.

## Reviewer Performance: Agreement by Rating

For the epochs that the group identified as HFOs:

The cyan bars illustrate that you were always at least as confident as the group average when you agreed with their consensus. Most commonly, you were 1 or 2 confidence points more certain than the group average.

The magenta bars show that you sometimes marked epochs as not containing HFOs when the group average indicated that they were present.

For the epochs that the group identified as non-HFOs:

The cyan bars illustrate that you were almost always as certain as the group average. Most commonly, you were 1 or 2 confidence points more certain than the group average. The lone cyan bar with a positive rating difference represents the lone epoch where you agreed with the group, but were less certain.

The absence of magenta bars shows that you never disagreed with the group

Overall:

This all suggests that while you rate both HFOs and non-HFOs confidently, you are in **general less likely** to call an event an HFO than the group on average.

## Epoch Presentation: Group Examples

*[The reviewer was shown each epoch for as long as the reviewer requested, and was read the corresponding statement]*

You will now be shown 7 stereotypical epochs. These epochs will depict diverse events, covering the range of the group’s HFO ratings.

[#1] This epoch was rated as **almost certainly** containing an HFO

[#2] This epoch was rated as **almost certainly NOT** containing an HFO

[#3] This epoch was rated as **likely** containing an HFO

[#4] This epoch was rated as **likely NOT** containing an HFO

[#5] This epoch was rated as **perhaps** containing an HFO

[#6] This epoch was rated as **perhaps NOT** containing an HFO

[#7] This epoch was effectively a toss-up, receiving low-confidence ratings for either HFO present or absent by all reviewers.

## Epoch Presentation: Reviewer vs. Group

Finally, you will now revisit 9 epochs, and will be given your rating and the group’s rating. Please spend as much time viewing each epoch as you feel is necessary. When you are ready, please prompt me to proceed to the next example.

*[The reviewer was shown each epoch for as long as the reviewer requested, and was read the corresponding statement]*

[#8] The group rated this epoch as containing an HFO, with a confidence of 2.4. Your rating was that the epoch contained an HFO, with a confidence of 4.

[#9] The group rated this epoch as not containing an HFO, with a confidence of 1.8. Your rating was that the epoch did not contain an HFO, with a confidence of 4.

[#10] The group rated this epoch as containing an HFO, with a confidence of 3.4. Your rating was that the epoch contained an HFO, with a confidence of 5.

[#11] The group rated this epoch as not containing an HFO, with a confidence of 3.8. Your rating was that the epoch did not contain an HFO, with a confidence of 5.

[#12] The group rated this epoch as containing an HFO, with a confidence of 2.7. Your rating was that the epoch contained an HFO, with a confidence of 5.

[#13] The group rated this epoch as not containing an HFO, with a confidence of 0.7. Your rating was that the epoch did not contain an HFO, with a confidence of 3.

[#14] The group rated this epoch as not containing an HFO, with a confidence of 4. Your rating was that the epoch did not contain an HFO, with a confidence of 5.

[#15] The group rated this epoch as not containing an HFO, with a confidence of 2.4. Your rating was that the epoch did not contain an HFO, with a confidence of 5.

[#16] The group rated this epoch as containing an HFO, with a confidence of 2.7. Your rating was that the epoch contained an HFO, with a confidence of 5.

## Conclusion

This concludes the feedback session. As mentioned earlier, you may choose to incorporate all, some, or none of this feedback into your future responses.

*[The next evaluation session was then opened for the reviewer]*

# Appendix 2 – Statistics

## Generalizability Studies

ANOVAs are widely used, and enable the statistical determination of whether one or more particular effects have a significant influence on the output variables (1). Generalizability theory – while still commonly used in pure statistics and education – has only been applied sparingly to the fields of neurology (2) and epilepsy (3). Rather than determining significance, it assesses the effects on the output variable exerted by one particular factor (dubbed the “object of measurement”), and how well these generalize to the universe of other factors (4-6). For example, if the object of measurement was a bunch of oranges, and if the output variable was their weight, then the measurements might be highly generalizable across the universe of industrial scales and grocery store employees, but not very generalizable across the universe of crude measuring devices and individuals who have never used any kind of scale. In effect, it can approximate measurement accuracy or interrater reliability (2, 3).

Another component of generalizability theory, called Decision studies, can estimate the levels of the other factors that would be necessary to obtain sufficiently generalizable measurements (5, 6). In the above example, it is clear that very few industrial scales or employees would be needed to obtain generalizable measurements of the oranges, but that many different crude measuring devices or untrained individuals would be required to isolate the intrinsic weight of the oranges from the measurement or user errors, thereby obtaining sufficiently generalizable measurements. This discrepancy can be captured using decision studies to project the number of measurement devices or individuals required in each case, providing a much more tangible point of comparison than the generalizability coefficients obtained in generalizability studies alone. The reader is directed to a textbook by Dr. Kline for a comprehensive overview of generalizability theory and decision studies (5), or to our previous work for details regarding the application of generalizability theory to HFO analysis (3).

## Confidence Intervals

Generalizability theory involves neither confidence intervals nor hypothesis testing. Rather, a single point estimate of the generalizability coefficient is computed in the generalizability study, and a single projection of the required sample size is computed in the decision study. Nonetheless, 95% confidence intervals were calculated for the variance components as below. As such, in addition to the point estimates, an “upper” and “lower” confidence limit was generated using variance components at the limits of their respective 95% confidence intervals. In particular, a “lower” confidence limit was obtained using the lower limit estimate for the object of measurement variance (i.e., the nested epoch variance) and the upper limit estimate for the other facets (e.g., the interaction between reviewer and nested epoch variance). Conversely, an “upper” confidence limit was obtained using the upper limit estimate and lower limit estimate for the object of measurement and other facets, respectively. This does not directly translate into a 95% confidence interval for the generalizability coefficient or decision study projection, but does provide a liberal “confidence interval” (CI) of these estimates to illustrate relative degrees of certainty.

## Statistical Analyses

ANOVAs were processed using the *UNIANOVA* function in SPSS, with Type III sum of squares. Generalizability theory variance components were calculated using the *remlVCA* function in the *VCA* package within R. Subsequently, 95% confidence intervals of the variance components were calculated using the *VCAinference* function within the *VCA* R package.

# References

1. Fisher RA. Statistical Methods for Research Workers. 5th ed. Crew FAE, Cutler DW, editors. Edinburgh: Oliver and Boyd; 1934.

2. Blood AD, Park YS, Lukas RV, Brorson JR. Neurology Objective Strutured Clinical Examination Reliability using Generalizability Theory. Neurology. 2015;85(18):1623-9.

3. Spring AM, Pittman DJ, Aghakhani Y, Jirsch J, Pillay N, Bello-Espinosa LE, et al. Generalizability of High Frequency Oscillation Evaluations in the Ripple Band. Front Neurol. 2018;9:510.

4. Kim SC. A Comparative Analysis of the Ratings in Performance Assessment Using Generalizability Theory and the Many-Facet Rasch Model. J Appl Meas. 2009;10(4):408-23.

5. Kline TJB. Psychological Testing: a practical approach to design and evaluation. Thousand Oaks, CA, USA: Sage; 2005. 185-200 p.

6. Brennan RL. Generalizability Theory. New York: Springer-Verlag; 2001.
